# Development and validation of an allele-specific PCR assay for genotyping a promoter and exonic single nucleotide polymorphisms of MGMT gene

**DOI:** 10.14440/jbm.2018.224

**Published:** 2018-06-07

**Authors:** Anuj Kumar Tyagi, Mary Boudal Khoshbeen, Patricia Huezo-Diaz Curtis, Chakradhara Rao S. Uppugunduri, Marc Ansari

**Affiliations:** CANSEARCH Research Laboratory, Department of Pediatrics, Faculty of Medicine, University of Geneva, Geneva, Switzerland; Onco-Hematology Unit, Department of Pediatrics, Geneva University Hospitals, University of Geneva, Geneva, Switzerland

**Keywords:** allele-specific PCR, exon, MGMT, rs2308321, rs113813075, genotyping

## Abstract

DNA repair protein *O*^6^-methylguanine-DNA methyltransferase (MGMT) specifically remove the methyl/alkyl group from the *O*^6^-position of guanine and restore the guanine to its normal form without causing DNA strand breaks. Relationship between MGMT activity and resistance to alkylating therapeutic agents is well established. Non-availability of simple, cost-effective and efficient methods of genotyping may hinder investigations on genotype-phenotype associations. No simple genotyping procedures such as allele-discrimination Taqman Assays were available for two genetic variations in MGMT gene that had previously demonstrated to be affecting its function and expression. These two variants were included to genotype in a clinical study (Clinicaltrail.gov ID: NCT01257854). Hence, the present study is aimed at developing, validating a rapid and simple allele-specific PCR method that genotypes exonic variant rs2308321 (c.520A>G) and a promoter variant rs113813075 (c.-459C>A) with standard PCR instruments. Web-based allele-specific (AS) primer design application called web-based allele-specific primer was used to design primers. Genomic DNA of lymphoblastoid cell line obtained from the Coriell repository with known genotypes were used to standardize the genotyping procedure. The PCR products were analyzed by 3% Agarose gel electrophoresis and by DNA Screen Tape assay with the Agilent 4200 TapeStation. The allele-specific PCR assay described here is a suitable strategy for efficient and reliable genotyping for difficult variants. This method offers cost-effective strategy for genotyping in clinical cohort studies provided positive controls established by Sanger sequencing are available for the variant.

## INTRODUCTION

*O*^6^-Methylguanine-DNA methyltransferase (MGMT) protein consists of 207 amino acids, which repairs the DNA damage at *O*^6^-methylguanine and is encoded by a single gene located on chromosome 10 at 10q26 [1]. The *MGMT* gene spans > 170 kb and contains 5 exons, 4 introns and the promoter region that lacks a TATA and CAAT box and includes the first exon and part of the first intron along with a minimal promoter and an enhancer element [2]. MGMT repairs *O*^6^-methylguanine by transferring the alkyl group to the sulfur of active cysteine residue in its active binding site. Following the transfer of the alkyl group to MGMT, the protein becomes inactive, ubiquitinated, and targeted for proteasomal degradation [3,4]. Mutagenic effects of alkylating agents are minimized by MGMT because if left unrepaired or mis-repaired, modifications introduced into the DNA may lead to an accumulation of mutations in the genomic DNA that may eventually lead to cancer development [5].

MGMT genetic variants may explain the differences in the MGMT enzyme activity and/or levels, which in turn may lead to reduced DNA repair capacity and thus increase cancer risk [5]. Polymorphic variants in the MGMT gene that affect the amino acid composition of the protein may affect the MGMT functions such as its ability to protect cells from carcinogenic and mutagenic alkylating agents along with its ability to alter the sensitivity of tumors to therapeutic methylating and chlorethylating agents [2]. A single nucleotide polymorphism (SNP) in exon 5 rs2308321 (c.520A>G or Isoleucine143Valine) has been linked to an increased risk of severe myelosuppression due to temozolamide, susceptibility to Hodgkins lymphoma, and myelodysplastic syndromes [6-8]. This SNP is important, as it is located in the active binding site in close proximity to the reactive alkyl-acceptor cysteine. This exonic region variant may be responsible for altering the geometry of the MGMT binding pocket by altering the amino acid sequence of the protein [2]. In addition, the amount of MGMT also determines the level of repair of DNA alkylation adducts and MGMT expression is highly regulated *via* promoter methylation [9]. Transcription factors and their interactions also affects the expression of MGMT because the MGMT promoter region has putative-binding sites for activating enhancer binding Protein 2 alpha (AP-2α) and a SNP, rs 113813075 (c.-459 C>A) is also located close to this binding site which may alter transcription factor interaction at this site [10]. Xu *et al*., recently described that a MGMT promoter haplotype that included this SNP (rs113813075) with c.-459 “A” allele had higher promoter activity compared to common reference haplotype with c.-459 “C” allele [10]. Thus, carriers of homozygous “AA” genotype at this locus may have higher levels of MGMT that can alter the sensitivity to alkylating agents.

For these reasons, we included these two variants along with other MGMT tagged genetic variants in a genotype-phenotype association study (Clinicaltrail.gov ID: NCT01257854). Allele discrimination assay (TaqMan) methodology was employed as the genotyping procedure in this study. However, it was discovered that no TaqMan assays were available for these two variants at the time of initiation of this study. One of the possible reasons for not having these assays could be that these variants are located within the GC rich regions making it difficult to design specific primers and probes. Hence, we evaluated available options for developing a simple and effective genotyping method for variants with this type of disposition. Different methods are available to carry out genotyping for detecting SNPs, some using automated systems developed for high throughput [11]. These methods are often reliant on expensive equipment and require relatively moderate to high development costs, and the marker assays generated are commonly not transferable between laboratories due to the diversity of assay technologies and infrastructure. A widely used method for detecting DNA sequence variants is allele-specific PCR in which one or both primers are designed to anneal at sites of sequence variation [12]. A simple method for genotyping could be applied easily in clinical investigations and association studies widely. Hence, in this report, we describe the development and validation of allele-specific PCR method for genotyping MGMT SNPs rs2308321 (c. 520A>G) and rs113813075 (c. -459C>A).

## MATERIALS AND METHODS

This study used a web-based allele-specific (AS) primer design application called web-based allele-specific primer (WASP) [13]. In particular, WASP makes use of different destabilizing effects by introducing one deliberate “mismatch” at the penultimate (second to last at the 3'-end) base of AS primers to improve the resulting AS primers [13]. For MGMT exonic SNP (c.520A>G), two reverse primers with a mismatch in the last 3’ nucleotides were designed for the variant of this polymorphism along with a common forward primer upstream of the polymorphic site with no mismatches. For variant (c.-459C>A) two forward primers with a mismatch in the last 3' nucleotides were designed along with a common reverse primer downstream of the polymorphic site with no mismatches. The allele-specific primer and the common primers were combined in two parallel PCR reactions (Primer sequences and commentary are listed in **Table 1**).

HPLC purified allele-specific primers were synthesized by microsynth (Switzerland) at a scale of 0.2 μmol. DNA ladders, MgCl_2_, dNTP’s were obtained from Fermentas Life sciences (Germany), and Taq DNA Polymerase gold and buffer were obtained from Promega (Madison, Wisconsin, USA). Genomic DNA was extracted from lymphoblastoid cell lines obtained from Coriell repository using Qiagen Blood Tissue extraction kit (Qiagen, San Jose, CA, USA) as per manufacturer recommendations. All extracted DNA samples were stored at **−**20°C until the analysis. The genotypes of lymphoblastoid cell lines were retrieved from 1000 genomes project database [14]. For each SNP, for each DNA sample, two PCR reactions were run in parallel. Each PCR reaction included one allele specific primer and a common primer. For example, to genotype rs2308321 two PCR reactions were run in parallel, one with primers c.520F and c.520T and a second reaction with primers c.520F and c.520C. The PCR reaction conditions for both the SNPs are given in **Table 2**. Necessary precautions were taken during the performance of each PCR reaction to prevent carry-over contamination. Both pre-and post-PCR reactions were performed in different rooms and all the reagents were handled using barrier filter pipette tips. Positive and negative controls were run during each PCR assay. Each PCR reaction of each sample was repeated on another day to rule out false negatives, and to test reproducibility. The PCR products were run in 3% agarose gels electrophoresis stained with SYBR^TM^ Safe (Invitrogen, USA) dye. The PCR products were also visualized using DNA Screen Tape assays (loading volume 2 µl) with the Agilent 4200 TapeStation (Agilent Technologies) as an alternative more rapid method and for double verification of product size.

The presence of a 192-bp and 134 bp PCR products indicated the presence of the alleles for the allele-specific primer used for rs2308321 and rs113813075, respectively. If amplification was detected in both allele specific reactions in a known homozygous positive control sample, and the signal strength was similar in both the reactions, this indicated that further optimization was required to differentiate between the sequences. Once optimized, the genotype was assigned based on the amplification, and if it was amplified in only one of the reactions then the sample was assigned as homozygous genotype for the allele specific primer used in that reaction. All the samples were re-amplified and were visualized on the gel to rule out false negatives. Though we used DNA of known genotypes for development and validation, Sanger sequencing of the PCR products was performed to confirm the genotype results. The list of lymphoblastoid cell lines and their respective genotypes for each SNP locus are given in **Table 3**.

## RESULTS AND DISCUSSION

The PCR was optimized by adjusting the concentration of each primer. The optimum annealing temperatures were determined using a gradient PCR. This is an important aspect of allele-specific PCR method to improve specificity of a reaction by identifying an optimal annealing temperature to avoid non-specific annealing of primers. Under optimized PCR conditions, the patterns of PCR product analyzed/visualized on the TapeStation and on agarose gel electrophoresis allowed the differentiation of the SNPs in order to determine whether the genotype is homozygous for the common allele, heterozygous or homozygous type for variant allele. The inclusion of samples with known genotype allowed us to observe the efficiency of the assay. Re-amplification and visualizing on the gel allowed us to rule out false negative results. The PCR assays produced reproducible results.

**Figure 1.**
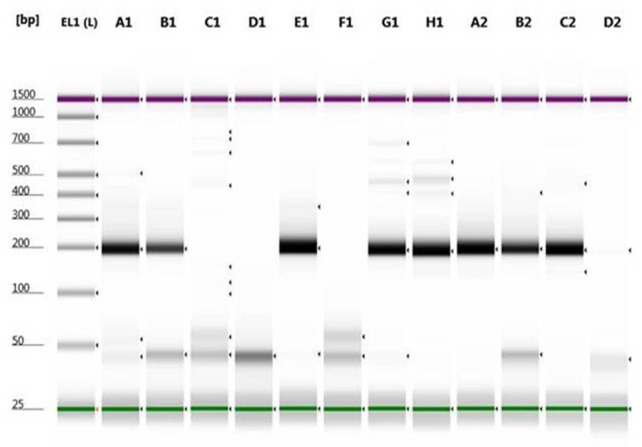
Genotyping of rs2308321 (c.520A>G) by allele-specific PCR using lymphoblastoid cell lines genomic DNA samples on TapeStation. Two separate PCR reactions were performed in parallel, one with primers c.520F and c.520C (amplify CC or GG allele) in lane A1, B1, C1, D1, E1 and F1 and a second with primers c.520F and c.520T (amplify TT or AA allele) in lane G1, H1, A2, B2, C2 and D2. A 192-bp band indicated the presence of the allele; amplification failure indicated absence of the allele. lane 1: electronic DNA ladder (EL1); lane A1 and G1: (NA-07034, GA); lane B1 and H1: (NA-7048, GA); lane C1 and A2: (N A-12762, AA); lane D1 and B2: (NA-10859, AA); lane E1 and C2: (NA-12763, GA); lane F1 and D2: Negative controls (H_2_O).

**Figure 2.**
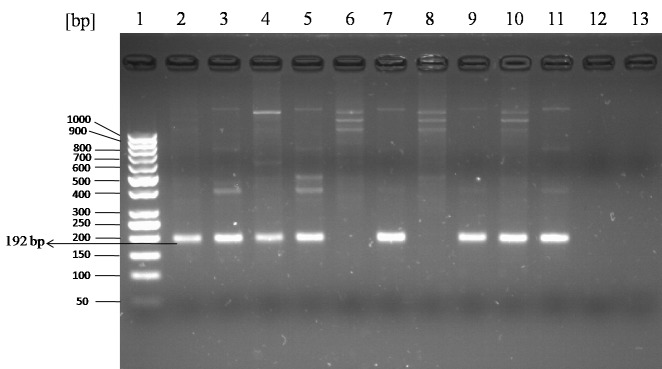
Genotyping of rs2308321 (c.520A>G) by allele-specific PCR using lymphoblastoid cell lines genomic DNA samples on 3% agarose gel electrophoresis. Two separate PCR reactions were performed in parallel, one with primers c.520F and c.520C (amplify CC or GG allele) in lane 2, 4, 6, 8, 10 and 12 and a second with primers c.520F and c.520T (amplify TT or AA allele) in lane 3, 5, 7, 9, 11 and 13 and run side by side on 3% agarose gel. A 192-bp band indicated presence of the allele; amplification failure indicated absence of the allele. bp: base pair; lane 1: 50 bp DNA ladder; lane 2 and 3: (NA-07034, GA); lane 4 and 5: (NA-7048, GA); lane 6 and 7: (NA-12762, AA); lane 8 and 9: (NA-10859, AA); lane 10 and 11: (NA-12763,GA); Lane 12,13: Negative controls (H_2_O).

**Figure 3.**
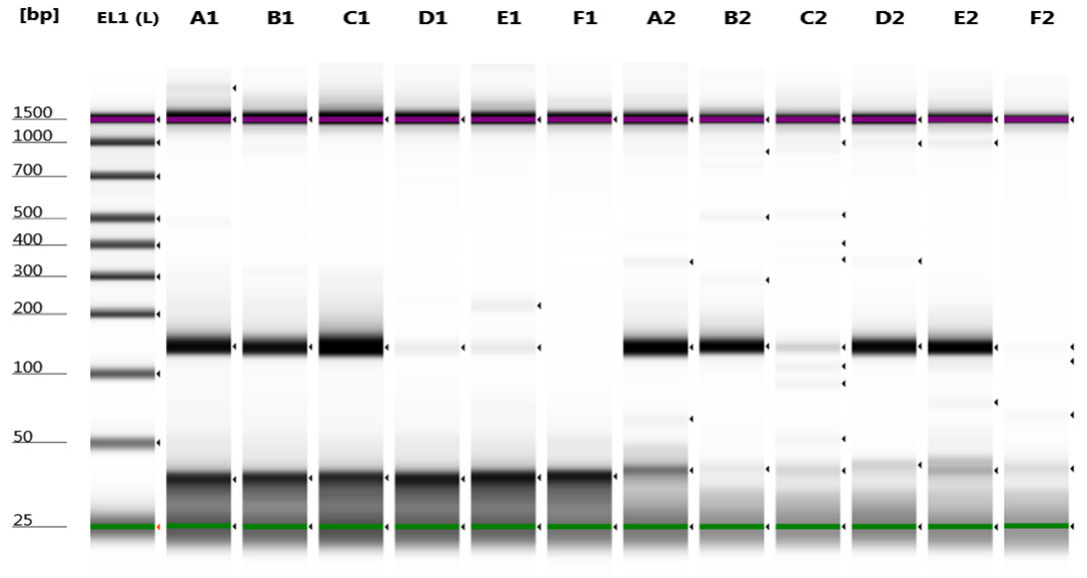
Genotyping of rs113813075 (c.-459C>A) by allele-specific PCR using lymphoblastoid cell lines genomic DNA samples on TapeStation. Two separate PCR reactions were performed in parallel, one with primersc.-459A and c.-459R (amplify AA allele) in lane A1, B1, C1, D1, E1, F1 and a second with primers c.-459C and c.-459R (amplify CC allele) in lane A2, B2, C2, D2, E2 and F2. A 134-bp band indicated the presence of the allele; amplification failure indicated absence of the allele. lane 1: electronic DNA ladder (EL1); lane A1 and A2: (NA-12004, CA); lane B1 and B2: (NA-12144, CA); lane C1 and C2: (NA-10847, AA); lane D1 and D2: (NA-6985, CC); lane E1 and E2: (NA-7000, CC); lane F1 and F2: negative controls (H_2_O).

**Figure 4.**
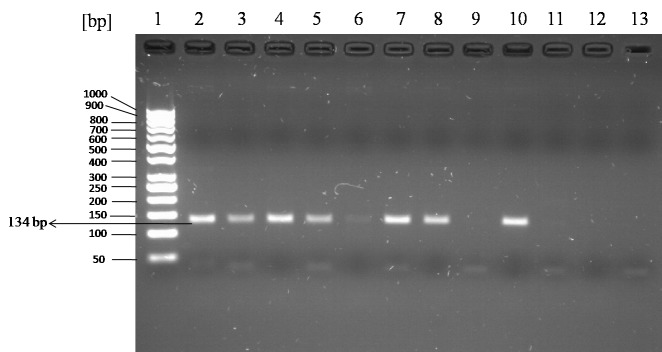
Genotyping of rs113813075 (c.-459C>A) by allele-specific PCR using lymphoblastoid cell lines genomic DNA samples on 3% agarose gel electrophoresis. Two separate PCR reactions were performed in parallel, one with primers c.-459C and c.-459R (amplify CC allele) in lane 2, 4, 6, 8, 10 and 12 and a second with primers c.-459A and c.-459R (amplify AA allele) in lane 3, 5, 7, 9, 11 and 13 and run side by side on 3% agarose gel. A 134-bp band indicated presence of the allele; amplification failure indicated absence of the allele. bp: base pair; lane 1: 50 bp DNA ladder; lane 2 and 3: (NA-12004, CA); lane 4 and 5: (NA-12144, CA); lane 6 and 7: (NA-10847, AA); lane 8 and 9: (NA-6985, CC); lane 10 and 11: (NA-7000, CC); lane 12 and 13: negative controls (H_2_O).

For rs2308321 and rs113813075, the minor allele frequencies reported from the 1000 genome project (http://www.1000genome.org) are 15% and 5.3%, respectively. The results obtained from the developed allele-specific PCR method were all consistent with genotypes of the samples taken form 1000 genome project database [14]. Although the genotypes results were known, they were already re-confirmed by Sanger sequencing of the purified PCR product amplified (**Fig**. **S1**). PCR products of each allele specific reaction for both rs2308321 and rs113813075 visualized on TapeStation (Agilent Technologies) and 3% Agarose gel after electrophoresis are shown in **Figure 1**-4.

There are several methods available for genotyping such as PCR-restriction fragment length polymorphism (PCR-RFLP), high resolution melting (HRM), Sanger sequencing, next generation sequencing and probe hybridization based techniques. PCR-RFLP has some disadvantages such as the necessity of an incubation period for enzymatic digestion by restriction endonuclease to separate the restriction fragments [15]. The other methods mentioned above are faster and easier to determine genotypes [16,17]. However, these methods are relatively time consuming in terms of data analysis and relatively expensive because they require the use of high technology instrumentation, software and expertise. The allele-specific PCR method developed in this study only requires basic equipment such as a conventional thermal cycler with gradient option and a gel documentation system, which are available in most laboratories. This method is easy to interpret and implement in clinical investigations from centers with limited infrastructure. This method does not use any specialized instrument, software or hybridization probes, whilst retaining test sensitivity and specificity with the inclusion of positive and negative controls. Moreover, the specificity of a PCR reaction can be improved upon introducing primers with a single nucleotide artificial mismatch introduced adjacent to 3' end (SNP site) [13]. This will increase specificity, especially when there is a pyrimidine base adjacent to the SNP site. Furthermore, in places where there is no problem with infrastructure facility, the TapeStation could be a convenient option for analyzing the allele specific PCR results in a short time with zero carryover contamination and **maximum efficiency**.

In conclusion, simple allele-specific PCR method described in this study is rather effortless, robust, and a reliable tool for genotyping variants with similar disposition as these two MGMT SNPs rs2308321 and rs113813075. Hence, this allele-specific PCR could be a strategy for genotyping SNPs where the assay is not available commercially and the research objective is specific for few candidate SNPs. Furthermore, it will enable researchers to carry out studies in places where lack of funding and equipment may be a factor. This methodology will allow the genotyping of patients in our clinical cohort.

## Supplementary Material

Supplementary information **Figure S1.** Electropherogram showing the confirmation of exonic SNP (rs2308321) and promoter SNP (rs113813075) genotypes by Sanger sequencing.
Supplementary information of this article can be found online at
http://www.jbmethods.org/jbm/rt/suppFiles/224.

## Figures and Tables

**Table 1.** Primers for rs2308321 (c.520A>G), rs113813075 (c.-459C>A) genotyping by allele-specific PCR.

Primer	Sequence (5'–3')	Commentary
c.520 F	TGCTGAGACATAGCTGACAC	Common forward primer
c.520 T	CACTCTGTGGCACGGGTT^a^	Reverse primer specific for allele T detection
c.520 C	CACTCTGTGGCACGGGTC^a^	Reverse primer specific for allele C detection
c.-459 C	GGCGCCGTCCTACGACTC^a^	Forward primer specific for allele C detection
c.-459 A	GGCGCCGTCCTACGACTA^a^	Forward primer specific for allele A detection
c.-459 R	TGAGGCTCTGTGCCTTAG	Common reverse primer

^a^Underlined bases indicate base specific changes in the primer sequence.

**Table 2.** PCR reaction conditions for both the SNPs.

Reaction components (25 µl)	MGMT : rs2308321	MGMT : rs113813075
MgCl_2_ (mM)	3.0	5.5
PCR Buffer	1 ×	1 ×
TaqDNA polymerase (U)	2.0	2.5
Primer concentration (each) **µ**M	0.2	0.28
dNTPs (mM)	1	0.8
DNA	50 ng	50 ng
Cycling conditions	Initial denaturation for 3 min at 95°C; followed by 40 cycles of 30 s at 95°C, 30 s at 57.6°C, and 30 s at 72°C; and final extension for 5 min at 72°C	Initial denaturation for 3 min at 95°C; followed by 35 cycles of 30 s at 95°C, 30 s at 59.4°C, and 40 s at 72°C; and final extension for 10 min at 72°C

**Table 3.** DNA samples used for PCR method standardization.

	MGMT : rs2308321		MGMT : rs113813075	
DNA samples used for method development and validation	NA07034	GA	NA12004	CA
	NA07048	GA	NA12144	CA
	NA12762	AA	NA10847	AA
	NA10859	AA	NA06985	CC
	NA12763	GA	NA07000	CC
